# Draft Genome Sequence of Non-*Vibrio parahaemolyticus* Acute Hepatopancreatic Necrosis Disease Strain KC13.17.5, Isolated from Diseased Shrimp in Vietnam

**DOI:** 10.1128/genomeA.00978-15

**Published:** 2015-09-17

**Authors:** Hidehiro Kondo, Phan Thi Van, Lua T. Dang, Ikuo Hirono

**Affiliations:** a Laboratory of Genome Science, Tokyo University of Marine Science & Technology, Tokyo, Japan; b Research Institute for Aquaculture No. 1, BAC Ninh, Vietnam

## Abstract

A strain of *Vibrio* (KC13.17.5) causing acute hepatopancreatic necrosis disease (AHPND) in shrimp in northern Vietnam was isolated. Normally, AHPND is caused by *Vibrio parahaemolyticus*, but the genomic sequence of the strain indicated that it belonged to *Vibrio harveyi*. The sequence data included plasmid-like sequences and putative virulence genes.

## GENOME ANNOUNCEMENT

Cultured shrimp have a high economic value. Shrimp production had been steadily increasing until 2011, when the outbreak of a new disease, called early mortality syndrome (EMS) or acute hepatopancreatic necrosis disease (AHPND), caused severe damage to the industry (1, 2). AHPND causes mortality of as high as 100%, and the causative agent of AHPND is a strain of *Vibrio parahaemolyticus*. The sequenced genomes of strains from Thailand and Mexico have a plasmid that might contain putative virulence genes (3, 4). Virulent strains can be identified by PCR using primers based on the virulence genes (5).

Because the virulence genes were on a plasmid, virulence might be transferred not only among *V. parahaemolyticus* strains but also to different bacterial species. In Vietnam, an AHPND strain, KC13.17.5, was isolated and identified to be *Vibrio* sp., but the 16S rRNA gene sequence showed that it was not *V. parahaemolyticus*. To further characterize this strain, its genome was sequenced.

Bacterial DNA was prepared according to Sambrook and Russell (6). A mate-pair library was generated from DNA using an Illumina Nextera XT DNA sample preparation kit. The library was sequenced using Illumina MiSeq and MiSeq reagent kit version 2 (300 cycles). Sequence data were assembled with CLC Genomics Workbench version 6.5.1, and then the scaffolds were analyzed on the RAST server (7). BLASTn searches of the genome were used to find sequences homologous to known plasmid-like and putative virulence genes (8).

The genome of the strain was assembled into 42 scaffolds. Annotation by the RAST server identified 5,288 genes. By further characterization, the closest neighbor of this strain was predicted to be *Vibrio harveyi* ATCC BAA-1116. A homology search using the predicted genes also showed the higher similarity to the genes of *V. harveyi* and *Vibrio campbellii* than those of *V. parahaemolyticus*.

We previously found that contig 4 of TUMSAT_D06_S3 (63 kbp) was highly conserved among 3 AHPND strains but was not conserved in 3 non-AHPND strains (3). This sequence is likely a plasmid. In the present study, some of the scaffolds, such as scaffold 21 (29 kbp) and scaffold 27 (16 kbp), were almost identical to contig 4 of TUMSAT_D06_S3. The putative virulence genes were identified and used to diagnose AHPND by PCR (5). Because scaffold 25 (3.8 kbp) was identical to the virulence genes, we speculate that this strain was made virulent by acquiring the plasmid.

Here, we sequenced an AHPND-causing species of *Vibrio* that was not *V. parahaemolyticus*. This strain also possesses the putative toxin genes and related plasmid-like sequences. The strain was closest to *V. harveyi*, meaning that the toxin genes could be transmitted to different *Vibrio* species. Further research on how the genes are transmitted across species is needed to prevent the spread of AHPND.

### Nucleotide sequence accession numbers.

The partial genome sequences of the strain have been deposited in DDBJ/EMBL/GenBank under the accession numbers BBXN01000001 to BBXN01000052. The raw data for the shotgun sequencing have been deposited in DDBJ Sequence Read Archive with accession no. DRA003689.
